# Prevalence of Insomnia in Various Industries and Associated Demographic Factors in Night-Shift Workers Using Workers’ Specific Health Examination Data

**DOI:** 10.3390/ijerph18136902

**Published:** 2021-06-27

**Authors:** Jihye Lee, Yeonpyo Hong, Weonyoung Lee

**Affiliations:** 1 Korea Occupational Safety and Health Agency, Occupational Safety and Health Research Institute, Ulsan 44429, Korea; neonsilver01@naver.com; 2 Department of Preventive Medicine, College of Medicine, Chung-Ang University, Seoul 06974, Korea

**Keywords:** shift work, night work, insomnia, night-shift Workers’ Specific Health Examination

## Abstract

This study aimed to investigate the prevalence of insomnia in various industries and the associated demographic factors. We searched the nationwide night-shift Workers’ Specific Health Examination (nsWSHE) data extracted in 2015 information on factors associated with insomnia, including sex, age, and the number of workers in the workplace. The prevalence of moderate insomnia in the total industry was 4.6%. Industries with a high prevalence of insomnia included the arts, sports, and recreation-related services industry, followed by the activities of household as employers industry, financial and insurance activities industry, human health and social welfare industry, and accommodation and food services activities industry. The prevalence of insomnia was higher in women. Age was a significant factor. In general, insomnia was highest among those aged ≥60 years. Insomnia was highest in small-sized companies with fewer than five employees compared to large enterprises with more than 1000 employees. This study is the first to analyze the prevalence of insomnia among night-shift workers who participated in the WSHEs. The findings of this study can be used to prioritize intervention policies for insomnia among night-shift workers.

## 1. Introduction

Night-shift workers are at increased risk of workplace accidents, angina pectoris, myocardial infarction, cerebrovascular disease, depression [1], sleep disorders, peptic ulcers, and breast and prostate cancer [2]. The International Agency for Research on Cancer (IARC) redesignated shift work as a probable carcinogen to humans (group 2A) in 2019, which provided limited evidence for breast cancer and prostate cancer [3]. Shift work sleep disorder (SWSD) refers to sleepiness while working night shifts and sleeplessness during sleeping hours [4]. The association between shift work and insomnia is well established [5,6,7,8,9,10,11,12,13,14,15,16,17,18]. Sleep disorders in night-shift workers can lead to fatigue, insomnia, and excessive sleepiness, resulting in decreased productivity, inefficient work outcomes, and accidents [19]. Moreover, insomnia is an important cause of workplace accidents and can also decrease quality of life due to fatigue and drowsiness on non-work days [4]. Doi (2005) reviewed 24 studies (13 non-shift and 11 shift studies among Japanese workers) identified by using MEDLINE and Japan Cetra Revuo Medicina [20]. The results revealed that the prevalence of insomnia and other sleep problems is 5–45% for non-shift and 29–38% for shift workers. Meanwhile, in a nationwide study of the general population of Korea, self-reported short sleep duration was defined as <5 h per day [21], and the insomnia prevalence was 11.7% in men and 15% in women.

A change in the circadian rhythm driven by the body clock located in the suprachiasmatic nuclei of the hypothalamus is one of the factors that induces insomnia [22]. The circadian time system influences the secretion of hormones, such as melatonin [23]. However, shift workers frequently undergo circadian misalignment by desynchronizing their sleep–wake and fasting–feeding cycle from the circadian time system. Recent experiments indicate that circadian misalignment has an adverse effect on metabolic and hormonal factors, such as circulating glucose and insulin. A decrease in the melatonin level and an increase in body temperature during the day can lead to a shorter sleep cycle and longer periods of wakefulness [24,25,26]. Cessation of shift work reduces the risk of insomnia [27]. However, some degree of night work is inevitable in the public sector. Therefore, measures to prevent the deterioration in health and quality of life associated with shift work are imperative.

Important factors associated with insomnia include sex and age [28,29,30,31,32], low level of education [33,34], and depression [35,36]. Environmental factors, such as the presence of partner and children [30,37] and alcohol consumption before sleep [38], can also contribute to insomnia.

After the Labor Standards Act was enacted in 1953, assessment of the work environment became mandatory in 1981, and the Workers’ Specific Health Examination (WSHE) was introduced. Currently, WSHE is an important means of preventing occupational diseases and protecting and promoting the health of workers. To prevent occupational disease, it is necessary to detect and manage health conditions early, since workers can be exposed to various hazardous materials at work. The Occupational Safety and Health Act stipulates that employers should provide periodic health examinations on workers in Korea. WSHEs are performed on workers exposed to hazardous materials that require management, such as benzene and formaldehyde. Currently, there are 178 hazardous materials for which a WSHE is required every 6 to 24 months. The WSHE includes medical surveillance and confirmation tests, which consist of history taking and blood tests, with the aim of early detection of disease. If suspicious findings are found in the primary test, a secondary test is performed. Clinical diagnostic examinations, such as ultrasonography, computed tomography, and neurobehavioral testing, may be performed if indicated.

The night-shift WSHE (nsWSHE) started in 2014 and was gradually expanded to all workplaces in Korea. The WSHE, including the nsWSHE, is a complete enumeration survey, and the results can be considered to be representative of Korea. In addition, the prevalence of insomnia among all Korean workers can be calculated using the WSHE analysis. However, there has been a lack of studies that analyzed the workplace factors affecting insomnia, such as the number of employees in the workplace.

This study sought to utilize the nationwide nsWSHE data to analyze the prevalence of insomnia among night-shift workers, using the Insomnia Severity Index (ISI) to compare night-shift workers from various industries. Additionally, we sought to analyze the demographic factors associated with insomnia in each of the industries that conducts night shift work.

## 2. Materials and Methods

### 2.1. Estimation of the Prevalence of Insomnia

The nsWSHE, managed by the Occupational Safety and Health Research Institute (OSHRI), was conducted nationally in 2015. According to the Korean Occupational Safety and Health Act, the definition of night work is divided into two categories. The first is a case of when eight consecutive night working hours (including the hours from 12:00 p.m. to 5:00 a.m., with an average of 4 per month for 6 months) are performed. The second is a case of when an average of ≥60 h of work is performed per month between 10:00 p.m. and 6:00 a.m. of the following day for 6 months. A survey of 598,366 night-shift workers, conducted as a part of the WSHE, were categorized into the major types of industry according to the Korea Standard Industry Classification.

In Korea, the use of the ISI score as the primary item in nsWSHE is stipulated. The ISI was developed by Morin et al. (1993) [39]. The ISI is the most commonly used screening test to evaluate insomnia. It consists of seven items: difficulty in falling asleep, difficulty staying asleep, problem of waking up too early, sleep satisfaction, daytime dysfunction, severity of perceived insomnia, and worry about sleep. Each item was scored from 0 to 4 points, and the scores for the individual items were summed. The ISI score was divided into four stages of insomnia: ISI 0–7 = not significant; ISI 8–14 = mild; ISI 15–21 = moderate; and ISI 22–28 = severe. An ISI score above moderate insomnia is defined as having insomnia. The ISI is used not only in shift workers but also in the general population. Park and Lee (2019) evaluated ISI and Epworth Sleepiness Scale (ESS) [40]. The exploratory factor analysis (EFA) and confirmatory factor analysis (CFA) of the ISI and ESS had sufficient validity and reliability to be used for occupational health examinations about shift work. The standardization and validation of the Korean version of the ISI (ISI-K) was done by Cho et al. (2014) [41]. Their study showed that the ISI-K total score has internal consistency as confirmed by a Cronbach’s alpha of 0.92 and adequate reliability with the item-to-total-score correlations (item-total correlations) ranging from 0.65 to 0.84. A cut-off score of 15.5 on the ISI-K was optimal for discriminating patients with insomnia. Therefore, we chose a score of ≥15 as a definition of insomnia.

The nsWSHE data were analyzed to estimate the prevalence of insomnia both in the total industry and in the different types of industries, according to the Korea Standard Industry Classification. The four industries with the highest night-shift frequency reported in the nsWSHE were manufacturing, human health and social welfare, business facilities management, business support services, and transportation and storage industry, accounting for 47.6%, 17.7%, 11.2%, and 10.3% of the total night-shift workers, respectively. Those four industries account for 86.8% of the total nsWHSE.

### 2.2. Factors Associated with Insomnia

To investigate the factors associated with insomnia in each industry, we assigned the dependent variable to be the presence or absence of insomnia, as defined by the ISI score. The demographic factors, such as sex, age, and number of employees, were assigned to be the independent variables. We analyzed the effects of age and number of employees on insomnia by dividing the group by sex into men and women. Ages were divided into five groups: under 30, 30–39, 40–49, 50–59, and over 60 years. Workplace size was divided into five groups based on the number of workers: <5, 5–49, 50–299, 300–999, and ≥1000.

The study protocol was approved by the Institutional Review Board of the Occupational Safety and Health Research Institute (approval number: OSHRI-2017-04).

### 2.3. Statistical Analysis

The prevalence of industry-specific insomnia was calculated as a percentage. Frequency analysis of insomnia was conducted according to sex, age, and number of employees among the industries in the WSHE using the χ^2^ test. In addition, a generalized linear model (GLM) was used to assess trends involving sex, age group, and number of employees in the workplace, and the occurrence of insomnia.

The level of statistical significance was set at *p* < 0.05. The data were analyzed using SPSS (version 21.0; IBM Corp., Armonk, NY, USA) and R version 3.5.1 (R Foundation for Statistical Computing, Vienna, Austria).

## 3. Results

### 3.1. Estimation of the Prevalence of Insomnia Using nsWSHE Data

The industry-specific distribution of insomnia among the 598,366 night-shift workers who completed the WSHE in 2015 is shown in Table 1. The prevalence of moderate insomnia in the total population was 4.6%, with 4.2% of men and 5.7% of women reporting insomnia.

Most workers belonged to the manufacturing (47.6%), human health and social welfare (17.7%), business facility management and business support services (11.2%), and transportation and storage (10.3%) industries. The prevalence of moderate insomnia was 4.5% in the manufacturing industry, 5.8% in the human health and social welfare industry, 3.9% in the business facility management and business support services industry, and 3.6% in the transportation and storage industry. Industries with a high prevalence of insomnia were the arts, sports, and recreation-related services (18.3%), activities of household as employers industry (7.4%), financial and insurance activities industry (6.2%), human health and social welfare industry (5.8%), and the accommodation and food service activities industry (5.6%).

### 3.2. Factors Associated with Insomnia

The factors associated with insomnia in men among night-shift workers in each industry were investigated (Table 2). In 12 industries, except for those that could not be analyzed due to the small number of subjects, the association between age group, number of employees in the workplace, and the presence of insomnia was examined. Age group displayed a significant trend in six industries. Insomnia was highest in those older than 60 years, followed by the <30, 50s, and 40s, and 30s age groups in the manufacturing and transportation industries. As age increased, insomnia increased in the construction industry. Insomnia was highest in those older than 60 years, followed by the 30s, 40s, <30, and 50s age groups in real estate activities industry. Insomnia was highest in those older than 60 years, followed by the 50s, 40s, <30, and 30s age groups in the business facilities management and business support services industry. Insomnia was highest in those older than 60 years, followed by the 40s, <30, 50s, and 30s age groups in the human health and social welfare industry. Insomnia was highest in those older than 60 years, followed by the 50s, 40s, <30, and 30s age groups in arts, sports, and recreation-related services industry. Insomnia was highest in those older than 60 years, followed by the 40s, <30, 50s, and 30s age groups in membership organizations, repair, and other personal services industry.

The trend for number of employees in the workplace was significant in seven industries. The odds of insomnia decreased as the number of employees increased in four industries. These were manufacturing, transportation and storage, information and communication, and human health and social welfare industries. Mostly, the prevalence of insomnia was highest in workplaces with less than five employees, followed by those with 5–49 employees, 50–299 employees, 300–999 employees, and ≥1000 employees. In the construction industry, insomnia was highest in workplaces with 50–299 employees, followed by those with 300–999 employees, 5–49 employees, and ≥1000 employees. The art, sports, and recreation-related services industry demonstrated the highest prevalence among workplaces with 50–299 employees, followed by those with 5–49 employees, ≥1000 employees, and 300–999 employees.

The factors associated with insomnia in women among night-shift workers in each industry were investigated (Table 3). In three industries, except for those that could not be analyzed due to the small number of subjects, the association between age group, number of employees in the workplace, and the presence of insomnia was examined. Age group displayed a significant trend. In general, as age increased, insomnia increased. However, in the human health and social welfare industry, the prevalence of insomnia was highest among workers older than 60 years, followed by those that were in the 40s, 50s, 30s, and <30 years age groups.

The trend for the number of employees in the workplace was significant in three industries. The odds of insomnia decreased as the number of employees increased in manufacturing, business facilities management and business support services, and human health and social welfare industries. The prevalence of insomnia was highest in workplaces with less than five employees, followed by those with 5–49 employees, 50–299 employees, 300–999 employees, and ≥1000 employees.

## 4. Discussion

This study aimed to estimate the prevalence of insomnia among night-shift workers and identify the factors associated with insomnia using nsWSHE data. Additionally, the demographic factors associated with insomnia in each industry conducting night-shift work were analyzed using the nsWSHE data.

In 2015, the prevalence of night-shift workers with more than moderate insomnia comprised 4.6% of all night-shift workers. Prevalence was 4.2% among men and 5.7% among women, indicating that insomnia is more common in women (Table 1). When the cut-off value was expanded to include mild insomnia, the prevalence of insomnia was 30.6% nationwide for night-shift workers (data not shown). We determined the ISI score using the same method employed in a previous study [42]. The prevalence of insomnia in 136 shift workers and 154 non-shift workers in the automobile parts manufacturing industry was 6.6% and 2.6%, respectively. Thus, the prevalence of insomnia was statistically lower in non-shift workers than shift workers. The prevalence of insomnia in shift workers in a study by Kim et al. (2008) was higher than that of shift workers in the present study.

Akerstedt et al. (2010) examined the effects of entering or exiting shift work on sleep and sleepiness using longitudinal data [27]. In comparison with constant day work, entering shift work from day work increased the risk of insomnia, and leaving shift work reduced the risk. The entering and leaving shift work has a considerable impact on sleep and alertness. Gold et al. (1992) conducted a hospital-based survey among 635 US nurses [7]. In comparison with nurses who worked only day or evening shifts, rotating nurses had more sleep cycle disruption. Yong et al. (2017) estimated self-reported sleep problems by job characteristics, including shift work status among US workers using the National Health and Nutrition Examination Survey [43]. They estimated sleep duration, sleep quality, and sleep-related activities of daily living (ADL). Though the overall prevalence of short sleep duration, poor sleep quality, impaired ADL score, and insomnia are 37.6%, 19.2%, 24.8%, and 8.8%, respectively, night-shift workers had the highest prevalence of 61.8%, 30.7%, 36.2%, and 18.5%, respectively, which was statistically significant.

The prevalence of insomnia among shift workers was different from that in the general Korean population. In a study by Cho et al. (2009), insomnia was defined as either difficulty getting to sleep or getting back to sleep after waking up at night. The prevalence of insomnia was 20.2% in men and 25.3% in women, which significantly increased with age [44]. The prevalence of insomnia in this study was higher among women, which is consistent with the findings of Cho et al., (2009). Marie and Foret (1999) examined insomnia by sex and age [28]. They defined sleep difficulty symptoms with five categories: difficulty falling asleep, difficulty staying asleep, difficulty getting back to sleep, early morning awakening, and hypnotic medication use. The result showed that women were more vulnerable than men in reporting difficulty of sleeping. The logistic regression analysis in terms of the probability of experiencing sleep difficulties for women compared to men showed that sex was also a significant predictor of sleep difficulties. Suh et al. (2018) reviewed sex differences found in insomnia [45]. They suggested the need to discuss treatment recommendations when working with women population at different stages of their life span because they are more vulnerable to insomnia.

In the study by Kim et al. (2013), self-reported short sleep duration was defined as <5 h per day [21]. The prevalence was 11.7% in men and 15% in women. These data were collected nationwide from the general population, but the definition of insomnia was different from that of our study, making it difficult to directly compare the findings with those of our study.

In the present study, the prevalence of insomnia was 4.5% among the manufacturing industry, 5.7% among the human health and social welfare industry, 3.9% among the business facility management and business support services industry, and 3.6% among the transportation and storage industry (Table 1). Studies have analyzed the insomnia of shift workers in various industries in Korea. Among 51 emergency medical doctors surveyed, the proportion of insomnia after starting night shifts was 35% [46]. Among subway workers, 34.8% of night-shift workers suffered from insomnia symptoms [47].

To compare the present and previous prevalence, Lauckhaupt et al. (2010) examined short sleep duration defined as <6 h per day among civilian employees from 2004 to 2007 [48]. In total, 29.9% of workers had short sleep duration. Although 24.2% of workers reported short sleep duration from 1985 to 1990, the prevalence of short sleep duration has been increasing. As for the trend of nurses, Saracoglu et al. (2020) surveyed the Pittsburg Sleep Quality Index (PSQI) among 208 nurses in Turkey in 2020 [49]. The average score was 7.1 ± 4.7, and the deterioration in sleep quality was found in 53.6% of the nurses. Similarly, Han et al., (2016) surveyed the PSQI among 2033 nurses in Harbin Medical University, China from April 2015 to November 2015 [50]. The average PSQI was 7.3 ± 3.6, and 42.9% had deteriorated sleep quality.

A study from Finland reported that 18.9% of male bus drivers had difficulty falling asleep, as did 18.8% of female cleaners, 18.0% of male teachers, 3.7% of male directors, and 4.9% of male physicians [5]. A cross-sectional WOLF (WOrk, Lipids, Fibrinogen) cohort study from Sweden showed that the prevalence of falling asleep unintentionally at least once a month was 7.0% among employees [51]. Another study showed that the prevalence of sleep disorders, defined as insomnia and excessive sleepiness in night workers and those working rotating shifts, was approximately 10% [52]. A study reported that 23.3% of shift workers on North Sea oil rigs had insomnia [53].

In this study, the prevalence of insomnia differed by age group among men, in which the number of subjects was large enough for analysis (Table 2). In general, insomnia was highest among men older than 60 years. In general, as age increased, insomnia increased among women (Table 3). However, in the human health and social welfare industry, the prevalence of insomnia was highest among workers older than 60 years, followed by those in the 40s, 50s, 30s, and <30 years age groups. Therefore, workers over the age of 60 years constitute the most vulnerable group for insomnia, and a management plan for this age group should be prepared. As in the general population, aging was found to be significantly associated with insomnia in 198 police shift workers in Korea [31]. The subjects, 90.4% of whom were men, were members of the Gyeonggi Provincial Police Agency, and the age groups were divided into 20s, 30s, 40s, and 50s. In the present study, insomnia decreased with age, which is inconsistent with the findings of Park and Choi (2010). However, data from subjects over 60 years of age and women were lacking in the study by Park and Choi (2010).

The prevalence of insomnia was highest in small-sized companies with fewer than five employees and lowest in large enterprises with more than 1000 employees. It can be interpreted that small-sized companies are vulnerable to not only accidents but also adverse health effects. For example, there were organizations dedicated to safety and health in large-scale enterprises but not in small and medium-sized companies. Therefore, in Korea, Workers’ Health Centers have been established to manage workers’ health for industries with <50 employees. There are few previous studies on the differences in insomnia according to workplace size. Instead, a small number of studies have examined insomnia in large enterprises or small- and medium-sized companies. Nakata (2011) investigated the effects of poor sleep characteristics on workplace injury among full-time male employees of small-and medium-scale businesses [54]. A total of 1891 male employees, aged 18–79 years (mean, 45 years), in 296 small- and medium-scale businesses in a suburb of Tokyo were surveyed by questionnaire from August 2002 to December 2002. Work hours and sleep characteristics, including daily sleep hours, subjective sleep sufficiency, sleep quality, and ease of waking up in the morning, were evaluated. The author suggested that attention should be paid to managing poor sleep and reducing excessive work hours to improve workplace safety.

Espie et al. (2018) investigated the relationship between insomnia symptoms and workplace productivity in a global manufacturing company [55]. In their study, employees from a US-based company were invited to participate in an online evaluation comprised of the sleep condition indicator (SCI) measuring symptoms of insomnia (a high score indicated better sleep). Among 2798 employees, 72% were male, and their mean age was 46.3 years (standard deviation (SD): 11.8). Sleep was the poorest among plant staff (SCI = 3.70 (SD: 2.73)), followed by retail staff (4.34 (SD: 3.02)) and office staff (4.95 (SD: 2.83)). Furthermore, the target workplace was a large corporation with about 2800 workers, and there were various occupational groups. There are often a variety of occupations in large enterprises, so the prevalence of insomnia can be lower than that of small- and medium-sized companies composed of a single or small number of occupations.

Although small- and medium-sized companies were analyzed in our study, the type of industry was limited. Most studies on night-shift work focus on employee health in large companies, primarily in the healthcare and transportation sectors. Pepin et al. (2018) stated that many night-shift workers work on their own or in small businesses related to services or food [56]. They focused on insomnia concerning night work in pastry production and sales. A telephone survey of night-shift workers was proposed to all employers and employees in the French pastry industry via their insurance health prevention company. A total of 1313 men and 1309 women aged 22–50 years were enrolled in the study. Enquiries related to total sleep time on workdays as well as frequency and duration of napping episodes were included in the survey. Of the respondents, 26.2% complained of chronic insomnia, especially women aged 45–54 years (31%). In our study, the highest prevalence of insomnia was 18.3% among arts, sports, and recreating-related services, and 5.6% of accommodation and food service activities workers suffered from insomnia. Especially, the proportion of <300 employees among the accommodation and food services industry was 69.7%, and this was categorized as a small- and medium-sized company.

This study had some limitations. First, the WSHE data analysis was a cross-sectional study. Longitudinal studies are necessary to establish causal relationships. Second, aside from sex, age, and the number of employees in the workplace, other factors that may affect insomnia were not investigated. Information on the type of shift work, such as 8 h shift, 12 h shift, 24 h shift, and shift direction, were not collected. Third, there has been controversy about the validity and reliability of the ISI score, which is the primary item of nsWHSE for shift workers. The strength of this study is that it is the first to estimate the prevalence of insomnia in Korean night-shift workers using nationwide data. These data are representative of the current work situation for night-shift workers in Korea. An additional strength of this study is that the association between sex, age, number of employees in the workplace, and insomnia in each industry was analyzed using nationwide data.

Shift work is a strong risk factor for insomnia, and cessation of night-shift work reduces the risk of insomnia [27]. However, some degree of night work is inevitable in the public sector. Insomnia in night-shift workers can lead to fatigue and excessive sleepiness, resulting in decreased productivity, inefficient work outcomes, and workplace accidents, including injury and even death [19]. Therefore, measures to prevent insomnia and deterioration in health and quality of life associated with shift work are imperative. A number of studies have evaluated countermeasures or interventions in shift workers and proposed treatments, which include chronobiotic interventions, such as light exposure, the use of melatonin, hypnotic agents, caffeine, CNS stimulants (amphetamine), and the wake-promoting agents (modafinil and armodafinil) [4]. The findings of this study can be used to prioritize interventions for insomnia in various industries.

## 5. Conclusions

The prevalence of moderate insomnia among night-shift workers was 4.6% in 2015, according to the nsWSHE data. There were differences in the prevalence of insomnia between different industries. Insomnia was higher among women than men. Age was also a significant factor. In general, insomnia was highest among those older than 60 years. The number of employees in the workplace was also significant, as insomnia was highest in small-sized companies with fewer than five employees compared to large enterprises with more than 1000 employees.

## Figures and Tables

**Table 1:** Frequency distribution of night-shift Workers’ Specific Health Examination in 2015 according to type of industry *.

Industry		*n*	%	Insomnia			
				None	%	Insomnia	%
A	Agriculture, forestry, and fishing	661	0.1	635	96.1	26	3.9
B	Mining and quarrying	1071	0.2	1023	95.5	48	4.5
C	Manufacturing	285,101	47.6	272,237	95.5	12,864	4.5
D	Electricity, gas, steam, and air conditioning supply	8151	1.4	7824	96.0	327	4.0
E	Water supply; sewage, waste management, materials recovery	2246	0.4	2158	96.1	88	3.9
F	Construction	13,555	2.3	13,154	97.0	401	3.0
G	Wholesale and retail trade	10,092	1.7	9778	96.9	314	3.1
H	Transportation and storage	61,864	10.3	59,617	96.4	2247	3.6
I	Accommodation and food service activities	5526	0.9	5215	94.4	311	5.6
J	Information and communication	5961	1.0	5645	94.7	316	5.3
K	Financial and insurance activities	834	0.1	782	93.8	52	6.2
L	Real estate activities	6691	1.1	6464	96.6	227	3.4
M	Professional, scientific, and technical activities	5129	0.9	4983	97.2	146	2.8
N	Business facilities management and business support services; rental and leasing activities	66,855	11.2	64,246	96.1	2609	3.9
O	Public administration and defense; compulsory social security	3184	0.5	3051	95.8	133	4.2
P	Education	395	0.1	381	96.5	14	3.5
Q	Human health and social welfare	105,674	17.7	99,592	94.2	6082	5.8
R	Arts, sports, and recreation-related services	6330	1.1	5172	81.7	1158	18.3
S	Membership organizations, repair, and other personal services	9015	1.5	8705	96.6	310	3.4
T	Activities of households as employers; undifferentiated goods and services-producing activities of households for own use	27	0.0	25	92.6	2	7.4
U	Activities of extraterritorial organizations and bodies	4	0.0	4	100.0	0	0.0
Total		598,366	100.0	570,691	95.4	27,675	4.6

* Severity of insomnia symptoms was assessed using the Insomnia Severity Index.

**Table 2:** Insomnia symptoms of each industry in men from the night-shift Workers’ Specific Health Examination in 2015 *.

Industry			Variable	Exp (B)	95% CI		*p* Value	*p* for Trend
C	Manufacturing (*n* = 223,688)	Age group, years	<30	1				<0.001 ***
			30–39	0.718	0.624	0.825	<0.001 ***	
			40–49	0.746	0.642	0.866	<0.001 ***	
			50–59	0.877	0.734	1.047	0.147	
			≥60	1.427	0.790	2.578	0.238	
		Employees, in workplace, *n*	<5	1				<0.001 ***
			5–49	1.239	0.554	2.770	0.601	
			50–299	0.939	0.430	2.047	0.873	
			300–999	0.897	0.409	1.967	0.786	
			≥1000	0.636	0.291	1.391	0.257	
D	Electricity, gas, steam and air conditioning industry (*n* = 8019)	Age group, years	<30	1				0.051
			30–39	0.680	0.411	1.126	0.134	
			40–49	0.593	0.361	0.974	**0.039**	
			50–59	0.482	0.292	0.795	**0.004**	
			≥60	0.600	0.110	3.264	0.555	
		Employees, in workplace, *n*	<5	1				0.194
			5–49	0.975	0.354	2.682	0.960	
			50–299	1.258	0.479	3.302	0.641	
			300–999	2.274	0.726	7.128	0.159	
			≥1000	0.517	1.387	0.516	3.726	
F	Construction (*n* = 12,848)	Age group, years	<30	1				**0.038**
			30–39	1.082	0.698	1.678	0.724	
			40–49	1.226	0.778	1.931	0.380	
			50–59	1.586	0.939	2.676	0.084	
			≥60	4.990	1.604	15.522	**0.005**	
		Employees, in workplace, *n*	<5	1				<0.001 ***
			5–49	0.774	0.234	2.563	0.676	
			50–299	1.001	0.309	3.239	0.999	
			300–999	0.922	0.279	3.047	0.894	
			≥1000	0.367	0.106	1.270	0.113	
G	Wholesale and retail trade (*n* = 6363)	Age group, years	<30	1				0.724
			30–39	0.882	0.542	1.435	0.614	
			40–49	0.989	0.568	1.721	0.968	
			50–59	0.849	0.445	1.621	0.620	
			≥60	2.969	0.458	19.244	0.254	
		Employees, in workplace, *n*	<5	1				0.210
			5–49	1.496	0.302	7.405	0.621	
			50–299	1.035	0.223	4.802	0.965	
			300–999	1.629	0.349	7.600	0.534	
			≥1000	0.998	0.204	4.867	0.998	
H	Transportation and storage (*n* = 59,181)	Age group, years	<30	1				<0.001 ***
			30–39	0.653	0.354	1.207	0.174	
			40–49	0.684	0.380	1.229	0.204	
			50–59	0.844	0.468	1.521	0.573	
			≥60	1.934	1.005	3.722	**0.048**	
		Employees, in workplace, *n*	<5	1				**0.005**
			5–49	0.895	0.226	3.546	0.875	
			50–299	0.898	0.241	3.352	0.873	
			300–999	0.637	0.725	0.191	2.754	
			≥1000	0.405	0.569	0.151	2.146	
I	Accomodation and food service activities (*n* = 3431)	Age group, years	<30	1				0.557
			30–39	1.082	0.751	1.559	0.672	
			40–49	0.917	0.596	1.412	0.694	
			50–59	1.276	0.694	2.347	0.433	
			≥60	4.338	0.511	36.840	0.179	
		Employees, in workplace, *n*	<5	1				0.968
			5–49	0.514	0.058	4.583	0.551	
			50–299	0.509	0.059	4.369	0.538	
			300–999	0.495	0.057	4.331	0.526	
			≥1000	0.459	0.051	4.136	0.488	
J	Information and communication (*n* = 5259)	Age group, years	<30	1				0.167
			30–39	0.641	0.420	0.979	**0.040**	
			40–49	0.843	0.541	1.316	0.453	
			50–59	0.950	0.568	1.589	0.845	
			≥60	0.796	0.253	2.509	0.697	
		Employees, in workplace, *n*	<5	1				<0.001 ***
			5–49	0.915	0.250	3.344	0.893	
			50–299	0.773	0.220	2.718	0.688	
			300–999	0.489	0.138	1.729	0.267	
			≥1000	0.403	0.113	1.443	0.163	
L	Real estate activities (*n* = 6281)	Age group, years	<30	1				**0.013**
			30–39	1.196	0.614	2.328	0.599	
			40–49	1.123	0.570	2.212	0.737	
			50–59	0.870	0.471	1.605	0.656	
			≥60	2.001	1.048	3.824	**0.036**	
		Employees, in workplace, *n*	<5	1				0.491
			5–49	0.199	0.018	2.215	0.189	
			50–299	0.234	0.021	2.561	0.234	
			300–999	0.177	0.016	1.968	0.159	
			≥1000	0.158	0.010	2.523	0.192	
N	Business facilities management and business support services; rental and leasing activities (*n* = 54,362)	Age group, years	<30	1				<0.001 ***
			30–39	0.979	0.787	1.217	0.849	
			40–49	1.261	0.964	1.649	0.090	
			50–59	1.612	1.221	2.127	<0.001 ***	
			≥60	3.779	2.746	5.198	<0.001 ***	
		Employees, in workplace, *n*	<5	1				<0.001 ***
			5–49	0.755	0.365	1.563	0.449	
			50–299	0.676	0.331	1.380	0.282	
			300–999	0.687	0.333	1.417	0.310	
			≥1000	0.699	0.335	1.461	0.341	
Q	Human health and social welfare (*n* = 20,125)	Age group, years	<30	1				<0.001 ***
			30–39	0.907	0.791	1.039	0.159	
			40–49	1.095	0.913	1.313	0.330	
			50–59	0.920	0.752	1.125	0.417	
			≥60	1.905	1.304	2.784	<0.001 ***	
		Employees, in workplace, *n*	<5	1				<0.001 ***
			5–49	0.458	0.194	1.082	0.075	
			50–299	0.424	0.182	0.984	**0.046**	
			300–999	0.375	0.161	0.873	**0.023**	
			≥1000	0.282	0.122	0.655	**0.003**	
R	Arts, sports, and recreation-related services (*n* = 3876)	Age group, years	<30	1				<0.001 ***
			30–39	0.674	0.541	0.838	<0.001 ***	
			40–49	1.101	0.850	1.427	0.467	
			50–59	1.675	1.137	2.469	**0.009**	
			≥60	1.926	0.583	6.363	0.283	
		Employees, in workplace, *n*	<5	1				<0.001 ***
			5–49	0.609	0.107	3.468	0.576	
			50–299	0.788	0.148	4.201	0.780	
			300–999	0.190	0.036	1.001	**0.050**	
			≥1000	0.270	0.051	1.423	0.123	
S	Membership organizations, repair, and other personal services (*n* = 5974)	Age group, years	<30	1				**0.050**
			30–39	0.661	0.412	1.061	0.086	
			40–49	1.088	0.613	1.931	0.775	
			50–59	0.800	0.455	1.406	0.438	
			≥60	1.579	0.766	3.256	0.216	
		Employees, in workplace, *n*	<5	1				0.170
			5–49	0.775	0.228	2.637	0.683	
			50–299	0.891	0.267	2.971	0.851	
			300–999	1.888	0.464	7.671	0.374	
			≥1000	0.387	0.071	2.110	0.273	

* Severity of insomnia symptoms was assessed using the Insomnia Severity Index; Bold values represent statistically significant; *** *p* Value less than 0.001.

**Table 3:** Insomnia symptoms of each industry in women from the night-shift Workers’ Specific Health Examination in 2015 *.

Industry			Variable	Exp (B)	95% CI		*p* Value	*p* for Trend
C	Manufacturing (*n* = 61,413)	Age group, years	<30	1				<0.001 ***
			30–39	1.234	1.085	1.402	<0.001 ***	
			40–49	2.713	2.263	3.254	<0.001 ***	
			50–59	3.218	2.541	4.076	<0.001 ***	
			≥60	3.055	1.398	6.677	**0.005**	
		Employees, in workplace, *n*	<5	1				<0.001 ***
			5–49	0.263	0.046	1.506	0.134	
			50–299	0.189	0.034	1.058	0.058	
			300–999	0.147	0.026	0.827	**0.030**	
			≥1000	0.151	0.027	0.847	**0.032**	
N	Business facilities management and business support survices; rental and leasing activities (*n* = 12,493)	Age group, years	<30	1				<0.001 ***
			30–39	1.414	0.910	2.196	0.123	
			40–49	1.890	1.259	2.837	**0.002**	
			50–59	2.149	1.462	3.158	<0.001 ***	
			≥60	2.627	1.591	4.339	<0.001 ***	
		Employees, in workplace, *n*	<5	1				<0.001 ***
			5–49	0.722	0.148	3.511	0.686	
			50–299	0.544	0.115	2.570	0.442	
			300–999	0.573	0.119	2.757	0.487	
			≥1000	0.295	0.062	1.412	0.126	
Q	Human health and social welfare (*n* = 85,549)	Age group, years	<30	1				<0.001 ***
			30–39	1.052	0.923	1.200	0.447	
			40–49	1.997	1.638	2.434	<0.001 ***	
			50–59	1.851	1.499	2.287	<0.001 ***	
			≥60	2.691	1.762	4.108	<0.001 ***	
		Employees, in workplace, *n*	<5	1				<0.001 ***
			5–49	0.521	0.241	1.128	0.098	
			50–299	0.509	0.239	1.086	0.081	
			300–999	0.485	0.226	1.041	0.063	
			≥1000	0.337	0.158	0.720	**0.005**	

* Severity of insomnia symptoms was assessed using the Insomnia Severity Index; Bold values represent statistically significant; *** *p* Value less than 0.001.

## Data Availability

Not applicable.
